# Prevalence, motivations, lifestyle preferences, and basic health behavior among 1,350 vegan, vegetarian, and omnivorous Austrian school teachers and principals

**DOI:** 10.3389/fnut.2025.1677900

**Published:** 2025-11-18

**Authors:** Katharina C. Wirnitzer, Derrick R. Tanous, Clemens Drenowatz, Gerold Wirnitzer, Manuel Schätzer, Gerhard Ruedl, Werner Kirschner

**Affiliations:** 1 Department of Sport Science, Leopold-Franzens University of Innsbruck, Innsbruck, Austria; 2 Department of Secondary Education, University College of Teacher Education Tyrol, Innsbruck, Austria; 3 Department of Pediatric Oncology and Hematology, Working Group Prevention, Integrative Medicine and Health Promotion, Otto-Heubner Centre for Paediatric and Adolescent Medicine (OHC), Charité—University of Medicine Berlin, Berlin, Germany; 4 Charité Competence Center for Traditional and Integrative Medicine (CCCTIM), Charité—Universitätsmedizin Berlin, Berlin, Germany; 5 Division of Sport, Physical Activity and Health, University of Education Upper Austria, Linz, Austria; 6 RockITLime, Stans, Austria; 7 SIPCAN—Special Institute for Preventive Cardiology and Nutrition, Elsbethen, Austria

**Keywords:** plant-based, nutrition, exercise, physical activity, sport, public health, prevention, health promotion

## Abstract

**Introduction:**

Few European and Austrian adults live a healthy lifestyle. As critical role models, school teachers and principals are highly influential for delivering basic health education to children and adolescents.

**Objective:**

This investigation aimed to analyze the underlying motivations and lifestyle preferences for diet type adherence among school teachers and principals and the associations with basic health behavior.

**Methods:**

The present study followed a cross-sectional design. School teachers and principals in Austria fill out an online questionnaire, with questions on anthropometrics, physical activity levels, dietary behavior, and alcohol and smoking consumption. Statistical analysis was conducted with ANOVA and chi-squared tests.

**Results:**

The final sample included 1,350 participants (409 males, 941 females) with an average age of 45.8 ± 11.4 years. Health (46.4%) was the most important reason for dietary choice and sports engagement, and lifestyle (pooled 81.7%) the predominant lifestyle preference across all dietary subgroups. Prevalence of vegan, ovo-lacto-vegetarian and omnivorous diet of school teachers and principals was 2.3%, 5.2%, and 92.5%, respectively. Females were more likely to follow an ovo-lacto-vegetarian diet (6.4% vs. 2.4%; *p* < 0.01) or vegan/ ovo-lacto-vegetarian (9.0% vs. 4.1%) than males. For total sample, no differences were found across the dietary subgroups considering leisure time physical activity, sports and exercise levels (88.7%; *n* = 1,197) and weekly engagement in sports (range: 2.9–3.3 days/week), the prevalence of daily fruit consumption (62.4%), alcohol intake (81.5%), or smoking prevalence (11.0%). Vegetable intake was significantly higher among ovo-lacto-vegetarians and vegans (92.9 and 93.5%, respectively; *p* < 0.01) than in omnivores.

**Conclusion:**

This is the first study to investigate the potential differences in basic health behavior among refined dietary subgroups (omnivorous, ovo-lacto-vegetarian, and vegan) in school teachers and principals. The findings indicate that basic diet type differentiation is the first step towards fundamentally healthy behavior, however, further action must be taken to achieve better health among school teachers and principals in Austria (more physical activity, sports and exercise, and fruit and vegetable consumption, less alcohol intake and no smoking).

## 1 Introduction

At the 2024 Summer Olympics in Paris, for the first time in history, over 60% of the food (33% in the Athletes’ Village restaurant, 50% for staff, 60% of the snacks on offer to the public) was plant-predominant, including vegan options (1–3). Overall, the 2024 Olympic and Paralympic Games aimed to reduce the carbon footprint by 50% and cater for the many vegan ovo-lacto-vegetarian Olympic athletes and spectators (1–3). Following the International Olympic Committee (IOC), the Union of European Football Associations (UEFA) hosted the 2024 European Football Championship in Germany as an event with ‘driving force for the sustainable development of German and European society’ concerning environmental protection and sustainability by incorporating a vegan and ovo-lacto-vegetarian food offering for players and fans in every stadium (4–7).

Considerable evidence supports the shift in populations towards healthful plant-predominant diets, which reduces or eliminates the intake of animal-originated products while maximizing advantageous impacts on human, environmental, and planetary health (8). The production of animal-based foods takes up 83% of all arable land worldwide but provides only 18% of all calories consumed worldwide (ratio 1:4 at the expense of animal products) (9). Avoiding meat and dairy products can therefore make the greatest contribution to resource conservation and reduce the CO2 footprint of food (75% less greenhouse gas emissions, 75% less land consumption, 54% less water consumption, 73% less water pollution, 66% less biodiversity loss, and more) (9–11). According to researchers of the Oxford University and the Intergovernmental Panel on Climate Change (IPCC), dietary behavior is probably the single biggest way to reduce your impact on planet Earth (i.e., not only greenhouse gas emissions but also global acidification, eutrophication, land, water use, and more) (9, 12–14). Poore and Nemecek emphasize that switching to a plant-predominant diet holds the greatest potential for protecting individual and global health and promoting human sustainability at all socio-ecological levels (9), including the containment of infectious diseases, epidemics and pandemics, NCDs, and the depletion of resources (especially global resources), along with other health measures (13, 14).

The determinants of health indicate that social circumstances, environmental pollutants, genetics, the healthcare system, and personal behavior are the major contributors to health outcomes (15, 16). Likewise, personal behavior is known to make the largest contribution with at least 40% influence over health (15, 16). Thus, the WHO has provided the leading modifiable, behavioral risk factors contributing to NCD development: tobacco and alcohol use, unhealthy diet, and insufficient PA (17). Recent findings have found that few European adults (5.8%) follow a healthy lifestyle (from four modifiable behaviors: PA, fruit and vegetable consumption, not drinking alcohol to excess, and not smoking) and even fewer Austrian adults (2.8%) (18). In contrast, however, over 92% of annual deaths in Austria (vs. 75% globally) are caused by underlying NCDs, which are also dominantly responsible for the leading causes of years lived with disability (e.g., 34.7% cardiovascular disease, 23.5% cancer) (19–22), with half of the global adult population projected to have obesity by 2035 (23).

Consistent with physical activity (PA) and sports/exercise levels (17, 24, 25), food and dietary decisions can make a definitive difference by the immune system response and prevention, considering the risk of moderate-to-severe disease progression and premature death (8, 26–30). However, well-planned and diligently implemented vegan and ovo-lacto-vegetarian diets are considered most beneficial to human health (31). Vegans are generally more health-conscious (regular PA, no or less alcohol and nicotine consumption) than ovo-lacto-vegetarians and mixed dieters. This results in the fact that, on average, vegans in particular are healthier (corresponding parameters are more often within the normal range, e.g., blood pressure, blood levels of lipids, glucose, cholesterol, etc.), have the lowest body weight and BMI, are also less likely to develop overweight and obesity, and are less likely to suffer from NCDs and chronic diseases than mixed dieters and ovo-lacto-vegetarians [31, pp. 97–115, 126, 279, 362–408]. The link between behavior and health is well established, also as health is linked to one’s educational and academic level (32–41). Thus, education plays an essential role in contributing to an individual’s health, interests and knowledge, and consciousness resulting in a specific health behavior (health-promoting or health-threating). Over the life course, higher education is shown to have advantages in terms of health and longer lifespan, including a greater likelihood to achieve a higher socio-economic status, stronger influence on social issues, higher income later on, better protection against unemployment, and effective participation in socio-political public agenda politics (32–41). Thus, regardless of socio-economic background, school, as a health-promoting living environment for all protagonists alike, i.e., pupils, teachers and principals, provides a particularly suitable setting for the development of healthy behavior (42–44).

School teacher and principal health is important to maintain and enhance with scientific evidence given their influential position in providing a safe and educational environment in schools (45). Likewise, school teachers and principals are highly influential role models for delivering health knowledge and competencies through health education to children and adolescents regardless of directly teaching health subjects (46–50). The basic health behavior that teachers and principals exemplify is naturally and seamlessly impressed on school pupils, as the school environment is vastly distinguishable from the home environment, with the opportunity of respected adults of the community leading the growth of children rather than the parents (46, 51–53). Health promotion as a healthy lifestyle, especially PA, sports & exercise paired with a healthy diet, is included in the state mandate (Austrian curriculum) for primary and secondary schools as a cross-curricular educational goal (54). Currently, limited research has been carried out on teacher (55, 56) and principal health (57), mostly concerning mental health or stress management in the school environment. Therefore, a gap in the literature exists on the relationship between PA, sports & exercise and diet types (especially vegan and ovo-lacto-vegetarian) in schools.

Previous studies have examined the lifestyle of teacher health behavior without explicitly analyzing diet types (55, 58–70). Likewise, the individual perceived level of healthy lifestyle was previously self-reported by the teachers themselves (55). Additionally, the importance of the PA levels with diet type (Healthy Eating & Active Living: HEAL) was previously suggested although no further examination of teacher behavior was carried out (58, 59, 61–65). To the best of the authors’ knowledge, no previous study has investigated diet type in connection with PA levels among a sample of Austrian adults working in the school setting. Therefore, *From Science 2 School* is the first study to investigate school teachers and principals following vegan and ovo-lacto-vegetarian diets in connection with PA, sports & exercise. The present study aimed to analyze the prevalence and motivations of teachers and principals at secondary schools levels I and II following distinct diet types in connection with basic health behavior. It was hypothesized that Austrian secondary school teachers and principals following vegan and ovo-lacto-vegetarian diets have advanced health behavior. In the following, the term “vegetarian” is used synonymously with an ovo-lacto-vegetarian diet, which includes dairy products, eggs, and honey, but excludes fish, meat and processed meat products.

## 2 Methods

The study *From Science 2 School* was conducted with a cross-sectional design and included an interdisciplinary approach. The Austrian Federal Ministry of Education, Science, and Research (Bundesministerium für Bildung, Wissenschaft und Forschung) approved this nationwide study (Department 1/7—School and University Sports). The study protocol (see at https://www.science2.school/en/#Publications) was approved by the ethical committees of the Federal Education Authorities in Austria (Burgenland, Carinthia, Lower Austria, Salzburg, Styria, Tyrol, Upper Austria, Vienna, and Vorarlberg); interested readers are kindly referred to the official website and previous publications.

### 2.1 Procedure and measures

The questionnaire was standardized and available for secondary school teachers and principals (middle school and high school levels) in Austria online (from October 28, 2019 to July,102,020) through an server-certificate encrypted website (SSL; LimeSurvey: 03.17.16; see: Supplementary material—data sheet 2 in the study protocol), and all study participation was anonymized. Before the teachers and principals volunteered to participate in the study, signed informed consent was acquired with the disclosure of detailed information on the study procedure. Study participation may have been ceased at any time without repercussion. The questionnaire consisted of five sections: personal information (Part 1; e.g., Your nationality? Your sex?), PA and sports/exercise (Part 2; e.g., What is your top reason for being physically active? What is your main sports activity?), dietary habits (Part 3; e.g., What is your current diet type? What is your top reason for your current nutrition?), general health (Part 4; e.g., What is the top factor for your health? Do you consider PA and sports/exercise to be health promoting?), and other related aspects (Part 5; e.g., What is your top leisure time activity? Do you smoke cigarettes?). Throughout the survey, control questions were incorporated to identify incoherent responses and possible conflicts to benefit the reliability of the dataset. The design of the survey was piloted at three schools prior to the implementation of the nationwide assessment, as required by the Austrian Federal Education Authorities.

The questionnaire was distributed across Austria (nationwide) and acquired sociodemographic data on age, sex, teaching level (middle and/or high school), living environment (urban or rural rural), employment status (full time or part time), anthropometrics (height, weight, and calculated BMI (kg/m^2^) and kind of diet (vegan, vegetarian, omnivorous). In addition, data was collected on (i) lifestyle interests (alcohol, smoking, engagement in PA, sports & exercise, dietary preferences (eating meat, vegetarian-kind), lifestyle preferences (sport/exercise, vegan/vegetarian lifestyle, other), (ii) dietary motivation (animal/environmental protection, heath, sport performance, tradition, taste/preference, no and/or other reason), (iii) dietary behavior (fruit and vegetable intake, level of fluids consumed, and most common fluids), (iv) PA and sports/exercise participation (leisure time activity, fitness club activity, days per week), (v) alcohol consumption, and (vi) smoking habits. Considering the reported dietary intake (any consumption of meat and/or fish/seafood, milk and/or milk products, eggs, honey, fruits, vegetables, grains, potatoes, or legumes; multiple answers possible) with the initial self-reported diet type, school teachers and principals were shifted to the corresponding dietary subgroups: omnivorous diet (intake of meat and other foods from animal sources, but also plant foods), vegetarian diet (no intake of red/white meats or fish/seafood; intake of eggs, dairy products, and honey), or vegan diet (no intake of foods or products from animal origin, 100% plant-predominant diet) (71, 72).

### 2.2 Participant recruitment

**Figure 1:**
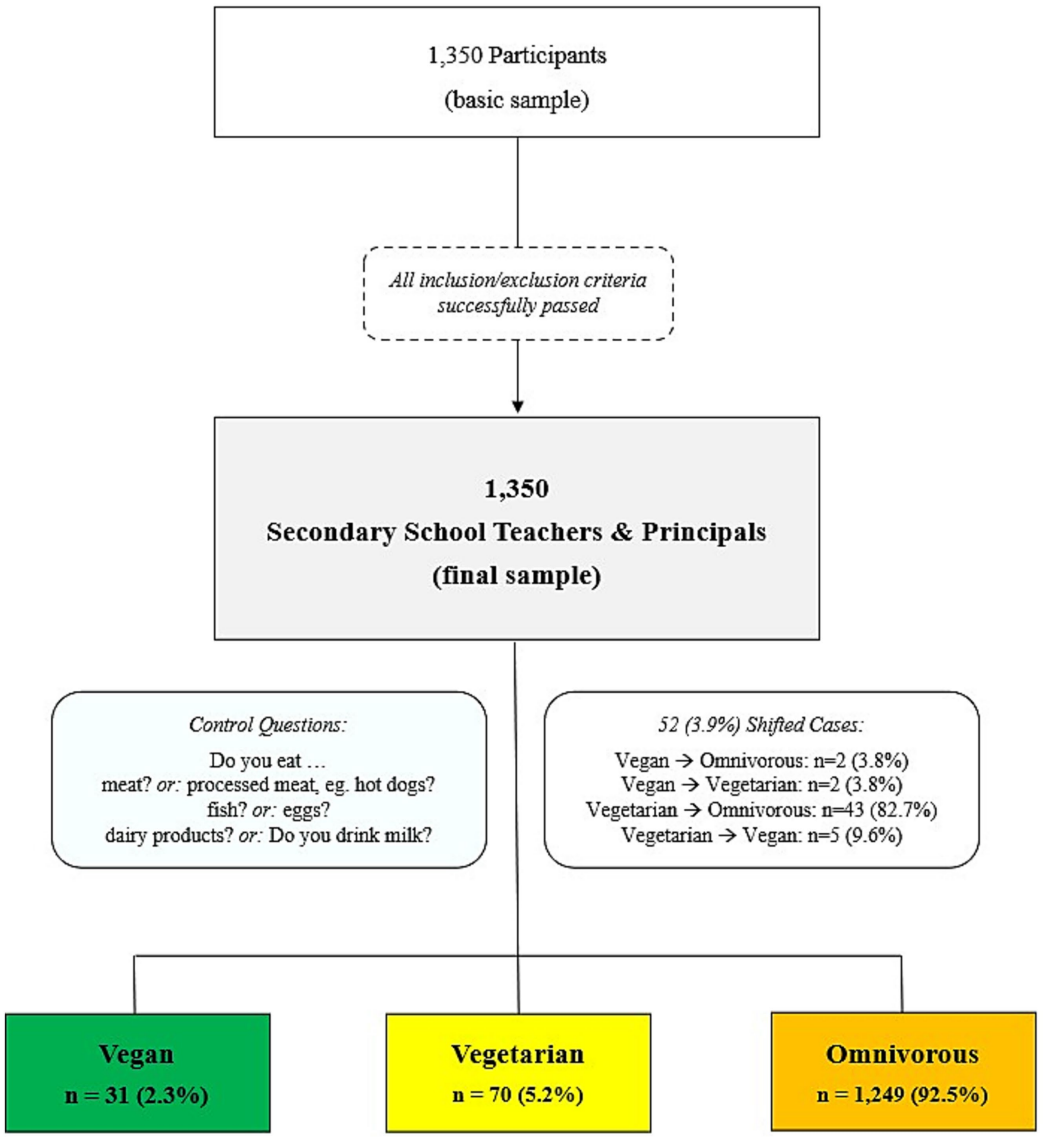
Flow of secondary school teachers‘and principals‘ study enrolment following vegan, vegetarian, and omnivorous, diets. Vegetarian—lacto-ovo-vegetarian.

The potential sample size included all teachers and principals (*N* = 89,243) of secondary (middle and high) schools nationwide in Austria. All secondary schools (*N* = 2,688) were invited to participate in the online survey by contacting the school principals by e-mail and asked to distribute and fill-in the survey (available in German) with a web-enabled device (e.g., via computer, smartphone, tablet). Signed informed consent was provided within the online survey interface, which was required to complete before survey participation was possible. The final sample included responses from 1,350 teachers and principals (1.5% of the eligible sample) at data closure (July 10, 2020). Figure 1 depicts the teachers´ and principals´ study enrollment.

### 2.3 Data clearance

After checking responses to various dietary control questions, the reported food consumed did not match the reported kind of diet in a total of 52 (3.9%) participants. Specifically, 2 vegan (3.8%) and 43 vegetarian (82.7%) participants were shifted to the omnivorous dietary subgroup. Further, 2 participants reporting a vegan diet (3.8%) were shifted to vegetarian while 5 participants reported eating vegetarian (9.6%) were shifted to vegan dietary subgroups. No participants reported implausible anthropometrics (such as a body weight < 20 kg, a height < 110 cm, or a BMI < 10 kg/m^2^ or > 50 kg/m^2^).

### 2.4 Statistical analysis

Prevalence and mean with standard deviation (SD) were used for the descriptive statistics. Significant effects (*p* < 0.001) were determined by post-hoc power analysis between dietary subgroups, including a yield of 98.2% statistical power for the given sample size (vegetarian prevalence 5.2% [4.1–6.5; 95%-CI], vegan prevalence 2.3% [1.6–3.2, 95%-CI]; two-sided 95%-CI, eta^2^ = 0.01). ANOVA and Bonferroni adjustment for post-hoc analyses were used to analyze differences in anthropometric characteristics between dietary subgroups. Associations between dietary subgroups and the prevalence of daily fruit and vegetable consumption, fluid intake, alcohol and nicotine consumption as well as participation in leisure time PA and sports/exercise were examined via Chi-square tests. Furthermore, differences across dietary subgroups in the number of days participants engaged in PA were examined via Kruskal-Wallis tests. Subgroup analyses were conducted for the total sample and sex, teaching level (middle school, high-school, middle and high school pooled), living environment (urban or rural) and employment status (full-time or part-time). SPSS 29.0 (SPSS Inc., IBM Corp. Armonk, NY, USA) was used to perform all statistical tests. The level of significance was set at *p* ≤ 0.01.

## 3 Results

A total of 1,350 secondary school teachers and principals were included in the present study. Most participants followed an omnivorous diet (92.5%; *n* = 1,249), while 70 (5.2%) and 31 (2.3%) participants followed a vegetarian and vegan diet, respectively. The majority of the omnivorous teachers and principals (91.1%; *n* = 1,138) reported that this has been their kind of diet for their entire life, while 5.7% (*n* = 4) reported that they have always consumed a vegetarian diet. Within the previous 5 years, 36.6% of vegetarian dieters and 64.5% of vegan dieters switched to their current diet from previously consuming an omnivorous diet.

Table 1 displays the distribution of participants across dietary subgroups based on the reported foods, which was used for subsequent analyses. Across the total sample, vegan participants were youngest with a mean age of 43.6 ± 10.5 years. Significantly more males reported an omnivorous diet (95.8%; *n* = 392), while the prevalence of vegetarian and vegan diet was higher in female participants (6.4%; *n* = 60; χ^2^ = 10.07, *p* < 0.01). There was no significant difference in BMI across dietary subgroups even though the prevalence of underweight was higher among participants with a vegan diet compared to their peers (χ^2^ = 17.78, *p* < 0.01).

**Table 1:** Anthropometrics and sociodemographic distribution of secondary school teachers/principals across dietary subgroups by sex, teaching level, employment status, and residence.

	Total	Vegan	Vegetarian	Omnivorous
	*N* = 1,350	2.3% (*n* = 31)	5.2% (*n* = 70)	92.5% (*n* = 1,249)
Age ± SD (years)	45.8 ± 11.4	43.6 ± 10.5	46.0 ± 11.4	45.8 ± 11.4
Sex^1^^,^^2^	Males 30.3% (*n* = 409)	1.7% (*n* = 7)	2.4% (*n* = 10)	95.8% (*n* = 392)
	Females 69.7% (*n* = 941)	2.6% (*n* = 24)	6.4% (*n* = 60)	91.1% (*n* = 857)
Height (cm)	171.2 ± 8.3	172.1 ± 11.6	169.5 ± 7.0	171.3 ± 8.3
Body Weight (kg)^3^	71.3 ± 14.6	68.2 ± 21.3	66.5 ± 10.9	71.6 ± 14.5
BMI (kg/m^2^)	24.2 ± 4.0	22.7 ± 4.3	23.1 ± 3.1	24.3 ± 4.0
Underweight^4^^,^^5^	2.6% (*n* = 35)	11.4% (*n* = 4)	2.9% (*n* = 1)	85.7% (*n* = 30)
Normal weight	63.0% (*n* = 851)	2.2% (*n* = 19)	6.0% (*n* = 51)	91.8% (*n* = 781)
Overweight	25.6% (*n* = 345)	1.4% (*n* = 5)	4.3% (*n* = 15)	94.2% (*n* = 325)
Obese	8.8% (*n* = 119)	2.5% (*n* = 3)	2.5% (*n* = 3)	95.0% (*n* = 113)
Teaching Level	Middle School 33.8% (*n* = 456)	2.2% (*n* = 10)	6.6% (*n* = 30)	91.2% (*n* = 416)
	High School 46.7% (*n* = 630)	2.1% (*n* = 13)	4.3% (*n* = 411)	93.7% (*n* = 590)
	Middle and High School Pooled 19.6% (*n* = 264)	3.0% (*n* = 8)	4.9% (*n* = 13)	92.0% (*n* = 243)
Employment Status	Full Time 77.9% (*n* = 1,052)	2.3% (*n* = 24)	4.7% (*n* = 49)	93.1% (*n* = 979)
	Part Time 22.1% (*n* = 298)	2.3% (*n* = 7)	7.0% (*n* = 21)	90.6% (*n* = 270)
Residence	Urban 37.7% (*n* = 509)	3.3% (*n* = 17)	6.7% (*n* = 34)	90.0% (*n* = 458)
	Rural 62.3% (*n* = 841)	1.7% (*n* = 14)	4.3% (*n* = 36)	94.1% (*n* = 791)

Vegetarian—lacto-ovo-vegetarian. Values are prevalences (%), total numbers, and mean ± standard deviation (SD). cm, centimeters; kg, kilograms. Significant difference (*p* < 0.01).

1 For omnivorous diet.

2 For vegetarian diet.

3 Between mixed and vegetarian diet.

4 Between mixed and vegan diet.

5 Between vegetarian and vegan diet (*p* < 0.01).

### 3.1 Dietary motives and lifestyle preferences

Health was the primary motive for the selection of any diet type. Almost half (48.4%; *n* = 15) of participants with a vegan diet reported health as the primary motive for their diet, followed by those with an omnivorous diet (46.9%; *n* = 586) and vegetarians (37.1%; *n* = 26). Animal welfare was the second most commonly mentioned motive among participants with a vegetarian (40.0%; *n* = 28) and vegan diet (29.0%; *n* = 9). Among those with an omnivorous diet, taste was the second most commonly mentioned primary motive (23.8%; *n* = 297) followed by tradition (10.2%; *n* = 127). Among vegetarians, taste was the third most commonly mentioned primary motive for their dietary choices (10.0%; *n* = 7), while environmental protection was the third most commonly mentioned motive (12.9%; *n* = 4) among those with a vegan diet. Food intolerances were reported by 14.0% (*n* = 189) of the participants.

Across the total sample, 70.7% (*n* = 954) of the participants considered engagement in exercise as a meaningful or cool lifestyle, and an additional 10.9% (*n* = 147) mentioned a lifestyle of a specific sports activity. A vegan diet and lifestyle were considered meaningful or cool by 7.4% (*n* = 100) and 2.4% (*n* = 32), respectively. While PA, sports & exercise remained the most commonly mentioned meaningful or cool lifestyle among those with a vegan diet (41.9%; *n* = 13), vegetarians reported their dietary choice most commonly as a meaningful lifestyle (51.4%; *n* = 36). A vegan diet was mentioned as a meaningful or cool lifestyle among 35.5% (*n* = 11) of participants with a vegan diet. Meat (4.5%; *n* = 56) and alcohol consumption (1.8%; *n* = 22) as a lifestyle were only mentioned among those consuming an omnivorous diet. Those with an omnivorous diet also mentioned the vegetarian diet (5%; *n* = 62) or vegetarian lifestyle (2.2%; *n* = 27) as a meaningful or cool lifestyle.

Table 2 displays an overview of dietary motives and lifestyle preferences of secondary school teachers and principals across subgroups, while full detail of dietary motives and lifestyle preferences for by diet type for sex, school level, residence and employment status are presented at Appendix Tables A.1 and A.2. (see Supplementary material).

**Table 2:** Dietary motives and lifestyle preferences according to diet type.

		Total	Vegan	Vegetarian	Omnivorous
		*N* = 1,350	2.3% (*n* = 31)	5.2% (*n* = 70)	92.5% (*n* = 1,249)
Dietary Motives	Health	46.4% (*n* = 627)	48.4% (*n* = 15)	37.1% (*n* = 26)	46.9% (*n* = 586)
	Animal welfare	4% (*n* = 54)	29% (*n* = 9)	40% (*n* = 28)	1.4% (*n* = 17)
	Taste/Preference	22.3% (*n* = 305)	3.2% (*n* = 1)	10% (*n* = 7)	23.8% (*n* = 297)
	Environment protection	1.6% (*n* = 22)	12.9% (*n* = 4)	5.7% (*n* = 4)	1.1% (*n* = 14)
	Other	6.4% (*n* = 87)	3.2% (*n* = 1)	1.4% (*n* = 1)	6.8% (*n* = 85)
Lifestyle Preferences	PA and sports/exercise	70.8% (*n* = 956)	41.9% (*n* = 13)	31.4% (*n* = 22)	73.7% (*n* = 921)
	Sports lifestyle	10.9% (*n* = 147)	3.2% (*n* = 1)	5.7% (*n* = 4)	11.4% (*n* = 142)
	Vegetarian diet	7.3% (*n* = 99)	3.2% (*n* = 1)	51.4% (*n* = 36)	5.0% (*n* = 62)
	Vegetarian lifestyle	2.4% (*n* = 32)	3.2% (*n* = 1)	5.7% (*n* = 4)	2.2% (*n* = 27)
	Vegan diet	1.1% (*n* = 15)	35.5% (*n* = 11)	4.3% (*n* = 3)	0.1% (*n* = 1)
	Vegan lifestyle	0.8% (*n* = 11)	9.7% (*n* = 3)	1.4% (*n* = 1)	0.6% (*n* = 7)
	Alcohol	5.9% (*n* = 80)	3.2% (*n* = 1)	4.3% (*n* = 3)	6.1% (*n* = 76)
	Smoking	1.3% (*n* = 18)	3.2% (*n* = 1)	NA	1.4% (*n* = 17)

Vegetarian—lacto-ovo-vegetarian. Values are prevalences (%) and total numbers. PA, Physical Activity; NA, no answer/not applicable.

### 3.2 Fruit and vegetable consumption

**Figure 2:**
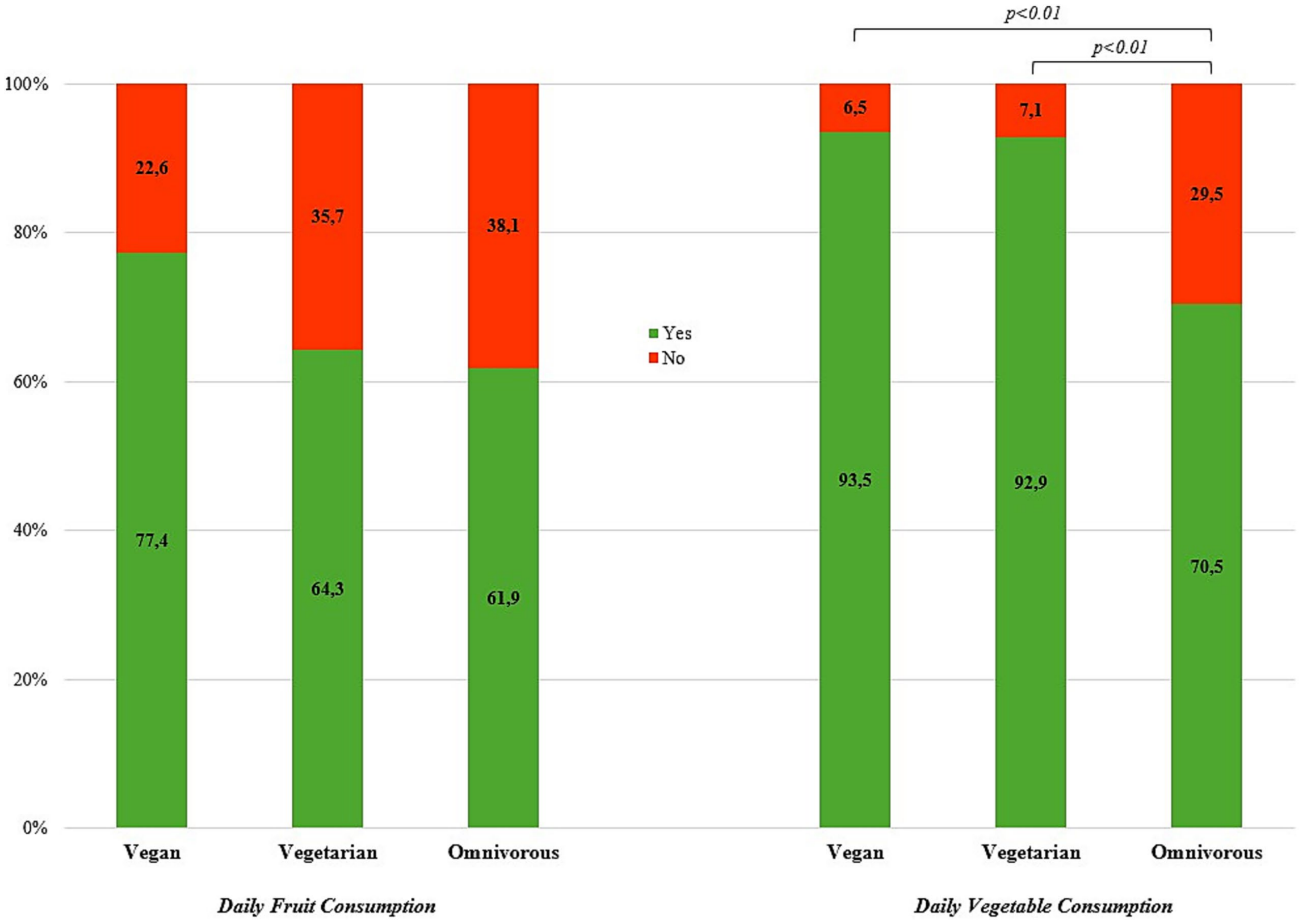
Prevalence (%) of daily fruit and vegetable consumption by diet type (*N* = 1,350). Vegetarian—lacto-ovo-vegetarian.

Among the total sample, 62.4% (*n* = 842) of the participants reported daily fruit consumption and 72.2% (*n* = 975) reported daily vegetable consumption. There was no difference in the prevalence of daily fruit consumption across dietary subgroups. The prevalence of daily vegetable consumption was significantly lower in omnivores (70.5%; *n* = 881) compared to vegetarians (92.9%; *n* = 65) and vegans (93.5%; *n* = 29; χ^2^ = 23.65, *p* < 0.01), while daily vegetable consumption did not differ between those reporting a vegan or vegetarian diet (Figure 2).

Male participants reported significantly less daily fruit and vegetable intake than females (χ^2^_fruit_ = 12.65, *p* < 0.01; χ^2^_vegetable_ = 28.52, *p* < 0.01). Further, part time teachers reported a higher prevalence of daily fruit and vegetable intake than their peer at fulltime employment (χ^2^_fruit_ = 10.69, *p* < 0.01; χ^2^_vegetable_ = 9.27, *p* < 0.01). Subgroup analyses further showed a higher prevalence of daily vegetable consumption in those with a vegan and vegetarian diet among full time teachers as well as secondary level I and II school teachers/principals (but at pooled subgroup). No difference was observed in the sex-specific prevalence of daily vegetable consumption across dietary patterns. Moreover, there was no difference in the prevalence of daily fruit consumption across kinds of diet in any subgroup. Table 3 shows the prevalence of daily fruit and vegetable consumption by dietary subgroups.

**Table 3:** Prevalence (%) of daily fruit and vegetable consumption by dietary subgroups.

Total (*N* = 1,350)	Daily Fruit Intake 62.4% (*n* = 842)			Daily Vegetable Intake 72.2% (*n* = 975)		
	Vegan	Vegetarian	Omnivorous	Vegan	Vegetarian	Omnivorous
	77.4% (*n* = 24)	64.3% (*n* = 45)	61.9% (*n* = 773)	93.5% (*n* = 29)	92.9% (*n* = 65)	70.5% (*n* = 881)
Sex						
Male	71.4 (*n* = 5)	50.0 (*n* = 5)	55.1 (*n* = 216)	100 (*n* = 7)	90.0 (*n* = 9)	61.0 (*n* = 239)
Female	79.2 (*n* = 19)	66.7 (*n* = 40)	65.0 (*n* = 557)	91.7 (*n* = 22)	93.3 (*n* = 56)	74.9 (*n* = 642)
Teaching Level						
Middle School	80.0 (*n* = 8)	70.0 (*n* = 21)	63.0 (*n* = 262)	90.0 (*n* = 9)	93.3 (*n* = 28)	69.2 (*n* = 288)
High School	76.9 (*n* = 10)	66.7 (*n* = 18)	61.4 (*n* = 362)	92.3 (*n* = 12)	96.3 (*n* = 26)	70.3 (*n* = 415)
Middle and High School Pooled	75.0 (*n* = 6)	53.8 (*n* = 6)	61.3 (*n* = 149)	100 (*n* = 8)	84.6 (*n* = 11)	73.3 (*n* = 178)
Residence						
Urban	76.5 (*n* = 13)	58.8 (*n* = 20)	61.1 (*n* = 280)	100 (*n* = 17)	91.2 (*n* = 31)	73.6 (*n* = 337)
Rural	78.6 (*n* = 11)	69.4 (*n* = 25)	62.3 (*n* = 493)	85.7 (*n* = 12)	94.4 (*n* = 34)	68.8 (*n* = 544)
Employment Status						
Full Time	70.8 (*n* = 17)	63.3 (*n* = 31)	59.7 (*n* = 584)	91.7 (*n* = 22)	93.9 (*n* = 46)	68.5 (*n* = 671)
Part Time	100 (*n* = 7)	66.7 (*n* = 14)	70.0 (*n* = 189)	100 (*n* = 7)	90.5 (*n* = 19)	77.8 (*n* = 210)

Vegetarian—lacto-ovo-vegetarian. Values are prevalences (%) and total numbers.

### 3.3 Fluids, alcohol, and nicotine consumption

**Figure 3:**
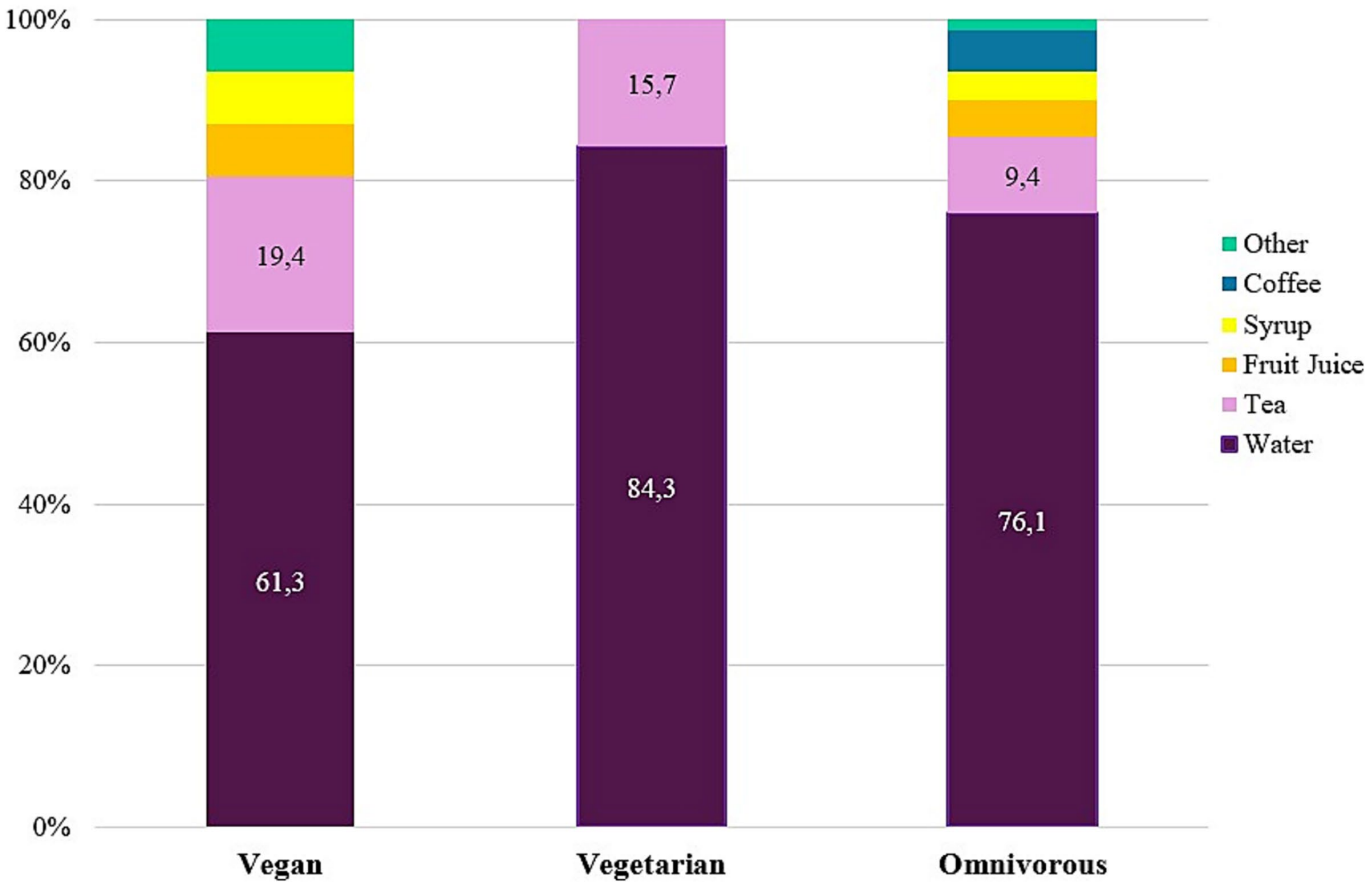
Prevalence (%) of most commonly consumed fluid by kind of diet (*N* = 1,350). Vegetarian—lacto-ovo-vegetarian.

Across the total sample 10.6% (*n* = 143) and 16.4% (*n* = 221) reported a fluid intake above 2.5 L/day and 2.0 L/day, respectively. There was no difference in level of fluid intake across dietary subgroups. Among the entire sample 76.1% (*n* = 1,027) of secondary school teachers/principals reported water as the most common drink, followed by tea (9.9%; *n* = 134), coffee (4.7%; *n* = 63) and fruit juices (4.4%; *n* = 59). Participants with a vegetarian diet only reported water (84.3%; *n* = 59) or tea (15.7%; *n* = 11) as their most preferred drink, and coffee was only the most preferred drink among participants with an omnivorous diet (5%; *n* = 62) (Figure 3).

**Figure 4:**
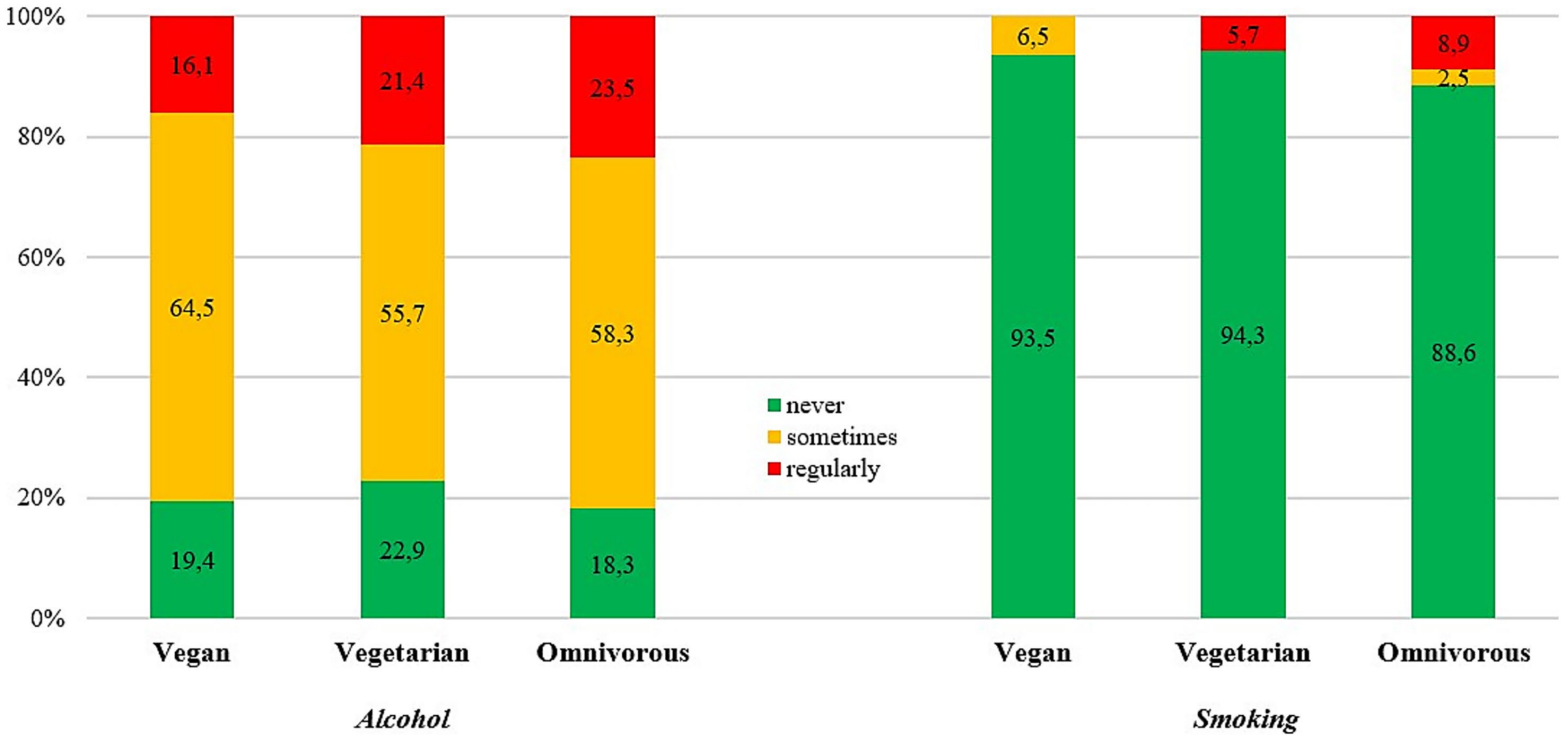
Prevalence of alcohol consumption and smoking by kind of diet (*N* = 1,350). Vegetarian—lacto-ovo-vegetarian.

Out of all secondary teachers and principals, 81.5% (*n* = 1,100) reported alcohol consumption and 11.0% (*n* = 149) smoked. Alcohol consumption and smoking prevalence did not differ significantly across dietary subgroups (Figure 4; Table 4).

**Table 4:** Prevalence (%) of alcohol consumption and smoking by kind of diet.

Total (*N* = 1,350)	Alcohol 81.5% (*n* = 1,100)			Smoking 11.0% (*n* = 148)		
	Vegan	Vegetarian	Omnivorous	Vegan	Vegetarian	Omnivorous
	80.6% (*n* = 25)	77.1% (*n* = 54)	81.7% (*n* = 1,021)	93.5% (*n* = 2)	92.9% (*n* = 4)	70.5% (*n* = 142)
Sex						
Male	85.7 (*n* = 6)	80.0 (*n* = 8)	86.7 (*n* = 340)	14.3 (*n* = 1)	20.0 (*n* = 2)	12.5 (*n* = 49)
Female	79.2 (*n* = 19)	76.7 (*n* = 46)	79.5 (*n* = 681)	4.2 (*n* = 1)	3.3 (*n* = 2)	10.9 (*n* = 93)
Teaching Level						
Middle School	80.0 (*n* = 8)	76.7 (*n* = 23)	78.6 (*n* = 327)	N/A	6.7 (*n* = 2)	13.5 (*n* = 56)
High School	76.9 (*n* = 10)	81.5 (*n* = 22)	85.4 (*n* = 504)	7.7 (*n* = 1)	7.4 (*n* = 2)	11.5 (*n* = 68)
Middle and High School Pooled	87.5 (*n* = 7)	69.2 (*n* = 9)	78.2 (*n* = 190)	12.5 (*n* = 1)	N/A	7.4 (*n* = 18)
Residence						
Urban	70.6 (*n* = 12)	85.3 (*n* = 29)	84.3 (*n* = 386)	11.8 (*n* = 2)	2.9 (*n* = 1)	12.2 (*n* = 56)
Rural	92.9 (*n* = 13)	69.4 (*n* = 25)	80.3 (*n* = 635)	N/A	8.3 (*n* = 3)	10.9 (*n* = 86)
Employment Status						
Full Time	83.3 (*n* = 20)	77.6 (*n* = 38)	82.7 (*n* = 810)	8.3 (*n* = 2)	4.1 (*n* = 2)	11.8 (*n* = 116)
Part Time	71.4 (*n* = 5)	76.2 (*n* = 16)	78.1 (*n* = 211)	N/A	9.5 (*n* = 2)	9.6 (*n* = 26)

Vegetarian—lacto-ovo-vegetarian.

### 3.4 PA, sports & exercise participation

Participation in leisure time PA, sports & exercise was reported by 88.7% (*n* = 1,197) of secondary school teachers/principals and 29.2% (n = 394) of them were members in sports clubs (Table 5). Compared to part-time employees full time employment was associated with higher leisure time PA (89.8% vs. 84.9%; χ^2^ = 5.65, *p* = 0.02) and club sports participation (30.8% vs. 23.5%; χ^2^ = 6.00, *p* = 0.01). There was no difference in leisure time PA by sex, living environment and employment status. Club sports participation, however, was significantly higher in male participants compared to female participants (39.1% vs. 24.9%; χ^2^ = 28.02, *p* < 0.01) as well as participants living in a rural environment compared to their peers living in an urban environment (31.4% vs. 25.5%; χ^2^ = 5.25, *p* = 0.02).

**Table 5:** Prevalence (%) of PA, sports & exercise engagement by dietary subgroups.

Total (*N* = 1,350)	Leisure Time Sports 88.7% (*n* = 1,198)			Club Sports 29.2% (*n* = 394)		
	Vegan	Vegetarian	Omnivorous	Vegan	Vegetarian	Omnivorous
	83.9% (*n* = 26)	88.6% (*n* = 62)	88.9% (*n* = 1,110)	29.0% (*n* = 9)	22.9% (*n* = 16)	29.5% (*n* = 369)
Sex						
Male	57.1 (*n* = 4)	80.0 (*n* = 8)	91.8 (*n* = 360)	28.6 (*n* = 2)	30.0 (*n* = 3)	39.5 (*n* = 155)
Female	91.7 (*n* = 22)	90.0 (*n* = 54)	87.5 (*n* = 750)	29.2 (*n* = 7)	21.7 (*n* = 13)	25.0 (*n* = 214)
Teaching Level						
Middle School	90.0 (*n* = 9)	86.7 (*n* = 26)	89.7 (*n* = 373)	40.0 (*n* = 4)	20.0 (*n* = 6)	31.0 (*n* = 129)
High School	76.9 (*n* = 10)	85.2 (*n* = 23)	88.6 (*n* = 523)	15.4 (*n* = 2)	25.9 (*n* = 7)	26.8 (*n* = 158)
Middle and High School Pooled	87.5 (*n* = 7)	100 (*n* = 13)	88.1 (*n* = 214)	37.5 (*n* = 3)	23.1 (*n* = 3)	33.7 (*n* = 82)
Residence						
Urban	82.4 (*n* = 14)	88.2 (*n* = 30)	89.1 (*n* = 408)	23.5 (*n* = 4)	23.5 (*n* = 8)	25.8 (*n* = 118)
Rural	85.7 (*n* = 12)	88.9 (*n* = 32)	88.7 (*n* = 702)	35.7 (*n* = 5)	22.2 (*n* = 8)	31.7 (*n* = 251)
Employment Status						
Full Time	79.2 (*n* = 19)	95.9 (*n* = 47)	89.8 (*n* = 879)	33.3 (*n* = 8)	24.5 (*n* = 12)	31.1 (*n* = 304)
Part Time	100 (*n* = 7)	71.4 (*n* = 15)	85.6 (*n* = 231)	14.3 (*n* = 1)	19.0 (*n* = 4)	24.1 (*n* = 65)

Vegetarian—lacto-ovo-vegetarian.

**Figure 5:**
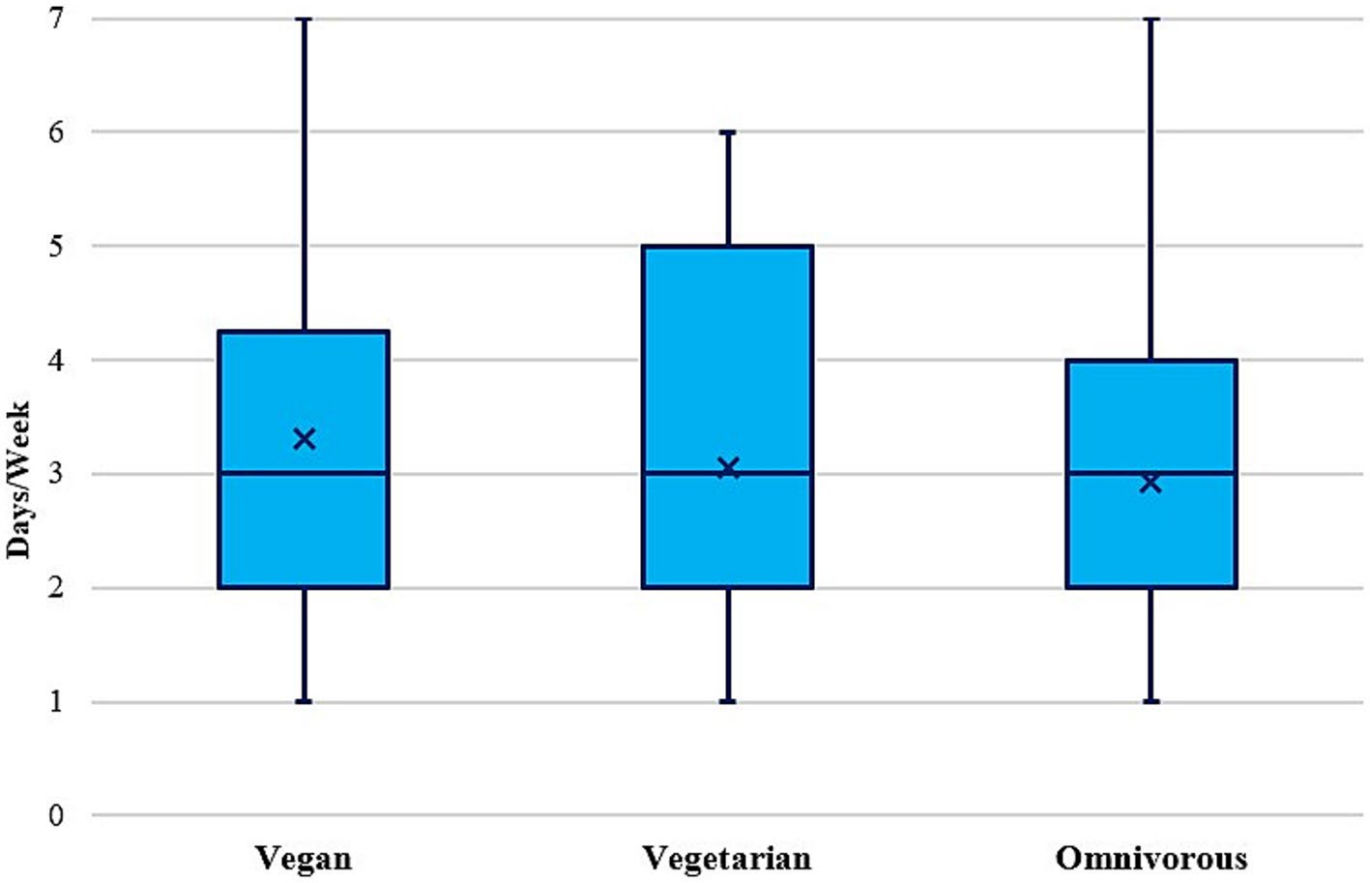
PA, sports & exercise participation (days/week) by diet type (*n* = 1,197). Vegetarian—lacto-ovo-vegetarian. Note. Values are displayed as median and interquartile ranges, including means (x).

Across the entire sample there were no differences in leisure time PA and sports/exercise or club sports participation across dietary subgroups, and—although vegans on average with 3.3 days/week were most active on a weekly basis (vs. 3.1 days/week in vegetarians and 2.9 days/week in omnivorous; Figure 5)—the frequency of PA and sports/exercise also did not differ by diet type. Similar results were observed in subgroup analyses, except for male participants regarding leisure time PA. In men, leisure time PA declined from those with an omnivorous diet to those with a vegan diet (χ^2^ = 11.56, *p* < 0.01) while no difference in club sports participation was observed.

## 4 Discussion

The current investigation from the *From Science 2 School* study aimed to assess the lifestyle interests, dietary motivations, and associations of key health behaviors among Austrian secondary school teachers and principals following vegan, vegetarian and omnivorous diets. The most important findings include: (1) the prevalence of each respective diet type show the majority of secondary teachers/principal following an omnivorous diet, with a vegan/vegetarian diet adherence similar to current reports; (2) significantly more males consumed an omnivorous diet and significantly more females consumed a vegetarian diet; (3) health is the main driver for a specific diet choice and PA, sports & exercise engagement and lifestyle were reported the predominant lifestyle preferences among school teachers/principals; (4) vegetable consumption was significantly greater among vegan and vegetarian subgroups; and (5) alcohol consumption and smoking prevalences were similar across dietary subgroups; and (6) although vegan teachers/participants are most active on a weekly basis, no differences were found regarding participation in leisure time PA, sports & exercise. Interestingly, despite vegan and vegetarian diet adherence among adults is linked to better health behavior and status due to higher health consciousness and interest [31: pp. 97–115, 126, 279, 362–408] as well as the established fact that health is linked to academic/educational level, the present investigation rejects the hypothesis that secondary level school teachers/principals following vegetarian (including vegan) diets have advanced health behaviors. The results include practical recommendations for upgrading school cafeteria (e.g., breakfast and lunch) options to include sustainable and healthy vegan snack and meal options daily with specific tailoring for promoting adult health (predominantly composed of a variety of whole foods low in fat, including whole grains, legumes, vegetables, fruits, nuts, and seeds combined with lightly processed foods; e.g., vegan meat alternatives, varieties of tofu, plant-based milks like almond or soy, plant-based yogurts, desserts, puddings, etc.) also suitable for vegan/vegetarian and omnivorous peers with lower environmental impact (9, 13, 14, 73).

### 4.1 Diet type prevalences, motivations, and sociodemographics

Typically, reports of vegetarian diets have been found to range around 7% of the global population (74). Whereas in German culture, vegetarian nutrition appears to be slightly less common, with known nationwide prevalences ranging up to 6% (75). The vegan diet, a special type of vegetarian nutrition, considering the absence of animal meat consumption as well as any product from animal origin (69, 70), is even less prevalent around the world (~2–3%) (74). However, background numbers reported 51% of European citizens, 51% of Austrian and 59% of German populations have reduced their meat intakes (76, 77), with at least 75 million (10%) Europeans adhere to a vegan or vegetarian diet (78). Austria currently has the highest fraction of people eating vegan (5% vegans, 5% vegetarians, 37% flexitarians) while Germany has the highest prevalence of flexitarian dieters (4% vegans, 6% vegetarians, 40% flexitarians) (76), with the prevalence of vegans and vegetarians in Germany having doubled during the COVID-19 pandemic (79). Thus, while approximately 9 in 10 teachers/principals reported eating a traditional mixed diet, the present investigation includes a similar prevalence of vegetarian dieters with vegan reports found slightly below the current average among Austrian school teachers/principals as compared to the general population. Interestingly, it was revealed that a considerable group of vegetarian dieters and most of the vegan dieters had switched to their current diet within the previous 5 years, indicating that many of these participants were rather novice among their dietary subgroups. However, it has been reported that (i) females are more likely to withdraw from the consumption of meat (or all animal products) and (ii) people with higher levels of education follow vegetarian nutrition to a greater extent, possibly due to underlying ethical considerations (e.g., that it is not a necessity to kill for survival or to maintain long-term sustainable health) (80). These findings may explain why the present veggie subgroups were more interested in the lifestyle of their diet as well as the motivations of the veggie dieters, which were mostly linked to health, environmental protection, and animal welfare. A recent report on contemporary eating trends found that especially health (47% vs. 49%), animal welfare (31% vs. 39%), and environment (27% vs. 30%) rank the top three motives in Austria, Germany, and across Europe (health: 38–57%; animal welfare: 13–40%; environment: 12–37%) (76, 77). Moreover, 8 in 10 teachers/principals of the present study reported PA, sports & exercise engagement and lifestyle as dominant lifestyle preferences with approximately 85% of omnivores reporting being active as most important.

Previous results from the WHO Health Behavior of School Aged Children (HBSC) on Austrian teachers (56, 57, 61), the WIENGS (Vienna’s network for health promoting schools), the Austrian Teacher and Principal Health Study (ATPHS) (55, 57, 61–65, 81), and the OECD TALIS (Teaching and Learning International Survey) (66, 67) reports, however, missed the importance of current nutritional trends, nicotine and alcohol consumption, and lacked the linkage of PA patterns with diet types.

In comparison, significantly more males were found to follow the omnivorous rather than a plant-predominant diet in the present investigation, which aligns with the previous literature on the topic, and is possibly due to an underlying cultural misappropriation of the link between meat and masculinity (80, 82). The Global Burden of Disease 2017 Diet Collaborators studied the health effects and overall impact of poor dietary habits on NCD mortality and found that the global consumption of processed meat, sodium, and red meat were markedly above the optimal levels (90%, 86%, and 18% greater, respectively), with men generally having higher intakes of healthy and unhealthy foods than women (83). Therefore, the sex difference between the omnivorous diet subgroup and vegetarian diet subgroup is likely one indication for explaining the difference in body weight between these groups. This finding is consistent with an Austrian report on teachers as a part of the WHO 2010 HBSC study (58, 59), including 60.6% of teachers having a normal BMI (vs. 63% in the current study), and overweight/obesity was much higher among males than females (40.6% vs. 14.7%). On the other hand, the underlying BMI difference may also be a reflection of replacing meat from the diet with a lower calorie plant food alternative (84). However, although vegans were found having the lowest BMI there were no significant differences in BMI across dietary subgroups, which is inconsistent with previous findings where adults consuming vegan diets were found to have the only normal BMI between the three groups (85). Additionally, the present findings on underweight in teachers are consistent with literature from the HBSC report (58, 59), as women (underweight prevalent in females only) tend to present such conditions more commonly than men.

### 4.2 Fruit and vegetable consumption

While daily fruit consumption did not significantly differ across the dietary subgroups, the vegans reported markedly higher daily intake than vegetarians and omnivores. In the EPIC-Oxford study, it was revealed that people eating vegetarian and vegan diets (i.e., more plant-predominant kinds of diets) consumed significantly more fruit (86). Therefore, an inconsistency was found with the previous research, although this tendency remains in the present findings. One explanation for the likely difference may be due to the rigorous definitions for each diet type, with the vegan dieters required to be eating 100% plant-predominant in the present investigation (71, 72) along with the higher health consciousness linked to generally higher academic/educational levels in teachers/principals. Whereas the classification for the omnivorous subgroup, on the other hand, must have indicated the consumption of animal meat. Therefore, the natural differences between these diet types allow for the heightened consumption of fruit on the vegan diet due to the exclusion of specific foods (meat, dairy products, eggs) and the replacement of calories with further plant-predominant foods. Since 1978, the positive health effects from plant-predominant diets are convincing (87) and frequently confirmed from further renowned studies on vegan and vegetarian diets (e.g., Adventist Health Studies (AHS) 1 and 2, EPIC-Oxford Study, Twin Nutrition Study (TwiNS)) as well as the connection with healthy longevity, better health outcomes, and greater health consciousness from healthier lifestyle patterns (28, 85, 86, 88–94). Subsequently, health outcomes revealed lower prevalences of NCDs and their risk factors among vegetarians and even more so among vegans (28).

This difference between the diet types was even more apparent considering daily vegetable consumption, as both the vegan as well as vegetarian dietary subgroup had significantly higher daily vegetables than the omnivorous subgroup. As fruit and vegetables are the primary components for a healthy diet, these foods should be consumed daily regardless of diet type, with five servings of fruit and vegetables recommended daily by the currently updated Austrian nutritional guidelines (95–97). In connection, less than daily fruit and vegetable consumption is considered to be poor dietary intake, which is known to be the cause of 15% of disability-adjusted life years (DALYs) and 22% (>1 in 5) deaths globally among adults (83); school teachers and principals in Austria as well as adults around the world are urged to at least consume 2 portions of fruit plus 3 vegetables every day (96, 97). Likewise, teachers are known to be major role models for imprinting the health behavior of their pupils (98, 99). The plant-predominant nature of the EAT-Lancet commission for the promotion of the planetary health diet is critically essential for all people to follow at a minimum for mitigating the imminent dangers of climate change (famine, disease, water shortages, forced migration, etc.) (100). In addition, typical meat-centered, high-caloric omnivorous dietary patterns containing red and processed meats are associated with a variety of health issues and adverse effects (e.g., cardiovascular disease, cancer, type 2 diabetes, all-cause mortality, etc.), including overweight/obesity as major risk factor amongst others (101–108). The negative effects of red meat on (all-cause and cardiovascular) mortality risk cannot be compensated for, even with the maximum intake from fruits and vegetables (109). However, reports on fruit and vegetable intakes showed 6 in 10 and 7 in 10 teachers/principals with respective daily consumption of the present sample, with better intakes among part-time employees and females. Regardless of the kind of diet, daily whole plant food consumption high in fiber and low in fat (>90% of daily calories) with considerable variety (legumes, whole grains, fruit, vegetables) may be increased to vastly promote health (110, 111). While the topic of the healthfulness and safety of vegetarian and vegan nutrition is still debated, often with an unenthusiastic or even prejudicial connotation (112–117), most dietitians (75%) lack knowledge on plant foods, protein, and especially whole food vegan diets and feel they did not receive adequate education (79%) (118).

A recent survey performed on 5,000 secondary school pupils aged 14–20 years initiated by the Austrian Pupils´ Representatives revealed that Austrian school children are generally interested in how nutrition affects physical health (87%), the impact of food on environment (71%), and the desire to receive more nutritional education in class during regular school time (82%) (119, 120). However, as by state mandate this learning content is already coped by school health promotion as the overarching teaching principle and educational goal across all grades (primary to secondary level II), with competence-oriented application relevant to teachers of all compulsory subjects enabling pupils to impart health knowledge in larger contexts (121). However, school sports as a starting point (health promotion and education considered as special task of school sports) and primarily the compulsory subject Physical Education has the leading role concerning holistic health concept contribution to the health promoting life organization even by dealing with such health topics (122–125). Therefore, and since not only dietitians and doctors lack basic health knowledge, teachers also need to be adequately educated and trained at university/college in order to be able to empower their pupils accordingly in the future (122–124).

### 4.3 Fluid intake, alcohol, and nicotine consumption

Adults in Europe are suggested to consume 2 or 2.5 liters of water per day (as plain water, other beverages, and/or within foods) for women and men, respectively (126). The vast majority of the present sample (73%) indicated less than adequate daily fluid consumption. However, the present participants did not consider the water contained in the foods they consumed as part of their daily fluid intake, which may have led to an adequate daily water intake, especially with foods high in water like fruits and vegetables (127). Considering that daily vegetable intake was higher among the vegetarian and vegan subgroups, this may have contributed to a better hydration status in these subgroups without revealing a difference in absolute fluid intake. About 3 in 4 teachers/principals (76.1%) of the total sample did, however, report water as the most common fluid consumed (with an additional water-based prevalence of approx. 10% from tea, adding up to a total of more than 8 in 10 participants reported daily water/tea intake), which is indicative of their alignment with basic health intuition (126). The omnivorous subgroup was the only group to consider coffee as the favored beverage, which may be related to the heightened awareness previously identified among vegetarians and vegans concerning the inessential substances they consume on a regular basis (128).

Interestingly, while most participants (approx. 8 in 10) reported alcohol intake with higher prevalence in full-time teachers/principals, and only a minor incidence of about 1 out of 10 reported smoking, no difference was found among the diet types concerning alcohol intake or smoking habits. For alcohol intake this finding is inconsistent with a previous large-scale, epidemiological study (AHS 2: vegetarians and vegans were found to drink dramatically less alcohol) and may be related to the present sample being schoolteachers and school staff of a European country, whereas previous findings were connected to a religious cohort in the USA (128). However, another study on Belgian adults found an association between vegetarian diets, alcohol consumption, and smoking habits, where the vegetarian (including vegan) subgroup was the least likely to consume either (129). The lack of difference found in the present investigation concerning alcohol consumption and smoking habits may also be related to the relatively small numbers of participants among the vegetarian (*n* = 70) and vegan subgroups (*n* = 31) with a considerable group of novice dieters (36.6% and 64.5%, respectively) compared to the total number in the omnivorous subgroup (*n* = 1,249). Also, it was previously found by the WHO HBSC study that teachers have a lower occurrence of smoking than the general population (12.6% vs. 23.3%) and most teachers were found to quit with older age (58, 59). The previous findings were in line with a study on German teachers indicating 20% smoking prevalence (69). Future research is suggested to include further analyses concerning inessential substance consumption among vegetarian and vegan dietary groups, especially considering subgroup analyses with participants adhering to their diet type for long-duration (e.g., >5 years, lifelong veganism/vegetarianism).

### 4.4 PA, sports & exercise levels, interests, and motivations

Nearly 9 out of 10 (or 89%) secondary school teachers/principals in the present study reported engagement in leisure time PA, sports & exercise (up to 92% in both vegan females and omnivorous males) with a big fraction of those (approx. one third) additionally being club sports members, too. A discrepancy with previous research was found regarding the similarity of PA levels of about 3 days/week across the dietary subgroups (85), with the vegans being most active of all dietary subgroups along with vegetarians on average being active at >3 days/week vs. omnivores at <3 days/week. In the present sample, the majority of teachers/principals (approx. 82%) considered an active lifestyle to be the most meaningful, which may partly explain the similarity between the participants´ PA levels. This finding may be related to PA, sports & exercise being the most frequently reported lifestyle interest among the vegan subgroup (41.9%). Likewise, about a third of Austrian teachers (32.5%) were previously found to engage in PA on at least three occasions per week (58, 59). The omnivorous subgroup, on the other hand, most frequently mentioned PA, sports & exercise as the top lifestyle interest (85.1%), however, this group performed the least weekly PA, sports & exercise. Previously it was found that 72% of teachers in Germany were engaging in regular sporting activity (69). This result may be connected to an increased interest for an exercise-focused (e.g., healthy) lifestyle but lacking the elevated energy levels to follow through. However, health was the top motivation among all dietary subgroups (46.6% overall), which may indicate that the present sample was more health conscious than the general population (130) possibly due to being more educated and holding professional roles within the context of the school setting. Previously, the Austrian HBSC report found that male and female teachers were more active than the general population (58, 59).

The overview of the present study shows healthier lifestyle behavior of Austrian secondary school teachers/principals (associated with BMI, PA pattern, alcohol intake, smoking) compared to the general population of Austria (58, 131, 132). This finding might be associated with the increased education and knowledge among teachers, particularly more individual attributes (including values, competencies, skills, certifications) and social conditions (e.g., networking, peer support) that aid implementing more scientifically supported healthy behavior (38, 55–59). According to an independent of the socio-economic background of children and adolescents, schools as a health-promoting environment are a particularly suitable setting for the development and training of healthy behaviors in crucial phases of life (42–44, 122). Likewise, previous data show the health of teachers is vital for the quality of teaching, including pupil learning outcomes (133), with the role of the teacher as a multiplier, being the leading factor of successful educational systems (134). In general, a close relationship exists between health, action competence, and personality development (52). Moreover, positive attitudes towards healthy lifestyle behavior is known to track over time (e.g., childhood to adulthood and old age) (135, 136). With regard to the UN SDGs #3—Good Health and Wellbeing and #4—High Quality Education as well as the UNESCO learning objective “cross-cutting competencies” (137–139), teacher health is not only a prerequisite but is essential for building successful societies; healthy teachers contribute positivity for the education and growth of healthy children, with a powerful influence on pupil lifestyle decisions (117, 140). Teacher health promotion is therefore not a “private matter” concerning the individual teachers themselves but a noteworthy contribution to the education system as a whole, reaching the general public, too (52), including public health benefits for nations like Austria.

Recent investigation from the health survey of 5,082 Austrian school headmasters and teachers (ATPHS action report) included no results on PA, sports & exercise, nutrition (fruit, vegetables), smoking, or alcohol consumption but urged the strategic importance of health promotion (62). In a recent report, teachers in an Austrian health study self-reported a high level of health (53). Likewise, the WIENGS study also lacked critical results on PA, sports & exercise, nutrition (fruits, vegetables), smoking, and alcohol due to a closer approach on mental health but highlighted the importance of health promotion at school for teachers and pupils (53, 59, 61, 81). Further, results from an overview on teacher health in Austria and Germany also failed to include findings on PA, sports & exercise, nutrition (fruits, vegetables), smoking, and alcohol with rising concern for the current mental health situation for teachers (63–65), and also the link pointed out by earlier research (70, 141). A recent study reported on the high-demanding nature of being a teacher, which may be related to a higher level of stress and hypertension (68, 142) although generally better health is found among teachers in Austria (63, 64, 143). However, before an improvement of the mental health situation among teachers may be realized, physical health still has much room for improvement, although generally better than the normal population (61, 142, 144). Results from the TALIS study also lacked the analysis of PA, sports & exercise, nutrition (fruit, vegetables) smoking, and/or alcohol consumption but found that teachers and headmasters are mostly satisfied with their professional roles (>93% vs. > 97%, respectively) (66, 67). Teachers are a valuable target group of research and important stakeholders in schools but have yet received little attention and help concerning health promotion (50, 145), and at the same time, are expected to take on even more functional and roles of importance in the school setting (49, 70).

### 4.5 Study limitations and strengths

Several limitations should be considered when interpreting the findings of the present investigation. The present study was developed as the first to examine differences in the motives, lifestyle preferences, and basic health behaviors of secondary school teachers/principals distinctly differentiated by vegan, vegetarian, and omnivorous diets in order to align with current trends towards more sustainable diets. While cross-sectional studies share the common limitation that no causality may be drawn from the results (146), the study design does allow for large samples to be accessed with particularly relevant themes based on previous literature to better elaborate the context of important areas for aiding future research with interventional designs or even randomized controlled trials (146). Importantly, the questionnaire of the present study was based on self-report data concerning health-related topics, which likely favored the occurrence of socially acceptable answers (possible reporting bias of, e.g., BMI, water intake, fruit, vegetable consumption). However, control questions were implemented throughout the questionnaire to identify inconsistencies for their removal previous to data analyses. In addition, the present sample appears to have provided highly reliable responses considering their respective professional roles (e.g., school teachers and principals, etc.) as pillars of society in connection with the finding of no study exclusions due to conflicting data sets on e.g., height and body weight, thus plausible calculated BMI. Moreover, with a population of of approx. 9 million, Austria has the largest vegan and also a large vegetarian population, respectively 5 and 5% (76, 77). The subjects in this investigation were predominantly female (*n* = 941; approx. 70%) also among vegan (>3:1-ratio) and vegetarian (6:1-ratio) dietary subgroups, which aligns with previous reports (80) and are in line with reports of females to be more health conscious and interested in following healthy, sustainable diets (147, 148). Likewise, study participation was voluntary, which may limit the results to the highly motivated teachers and principals (e.g., possible selection bias).

A major strength of the present investigation is the basis of the study on Austrian secondary school teachers and principals, as influential role models of favorable health behavior for society, including a large sample (1.5% of the total: 89,243 nationwide population). The present sample did, however, include a sex difference with a predominance of female vs. male participation (2:1 ratio) and more females in the vegan/vegetarian subgroups (5–6 times). While this finding may appear to increase bias, this is rather trivial considering the approach of the cross-sectional design. Additionally, while the results of the omnivorous dietary subgroup were based on a large and representative sample (power analysis revealed the minimum sample required was *n* = 984; see study protocol at https://www.science2.school/en/#Publications), it is still difficult to enroll a sufficiently large sample of vegans and vegetarians to participate in a study. Thus, although comparable (7.7% pooled) to current reports available on sustainable diet trends among the Austrian population, the respective numbers of vegan and vegetarian dieters in the present investigation were relatively low with a high prevalence of rather novice vegans (64.5%). The present study is the first to overcome the lack of knowledge and bridge the gap in current literature related to adult populations with higher academic level within the educational setting and also to consider the connection of diet type with basic health behaviors among school teachers and principals. Thus, the findings may be especially relevant for providing a basis for the promotion of future health among Austrian teachers, principals, superintendents, and other school staff as well as the close relationship with school pupils, their parents/guardians, and other similar socio-cultural backgrounds. In addition, future interventions or randomized controlled trials are recommended to investigate vegan dietary interventions in schools, primarily targeting the population of school teachers and principals.

## 5 Conclusion

The present investigation of secondary school teachers and principals health behavior as part of the *From Science 2 School* study revealed novel findings concerning the connection of diet type (vegan, vegetarian, omnivorous) to contribute to close the gap in teacher reports available. The present investigation found no broad advancement of health behavior among vegan/vegetarian dieters as compared to the general omnivorous dieters (PA behavior, fruit consumption, alcohol intake, or smoking habits), which may be due to the generally higher health interest and consciousness of teachers since health is linked to educational level. Higher health consciousness from a fundamental basic education and of further education among the present population of school teachers and principals may delineate the lack of differences between diet types and BMI in the present investigation. Additionally, health was the most important reason for dietary choice, sports engagement, and the predominant lifestyle preference across all dietary subgroups. School teachers, principals and pedagogues are highly valuable role models and multipliers for promoting the health situation of future generations. Thus, every effort (personal, scientific, school policy, governmental support, etc.) should be made to prioritize improving the health behavior of teachers, principals and pedagogues for benefitting the public health in Austria as well as similar nations around the world.

## Data Availability

The datasets presented in this article are not made publicly available due to data protection and security laws. Requests to access the datasets should be directed to katharina.wirnitzer@uibk.ac.at.
