# Supplementary material for: Prevalence, motivations, lifestyle preferences, and basic health behavior among 1,350 vegan, vegetarian, and omnivorous Austrian school teachers and principals
Source: Front Nutr. 2025 Nov 18;12:1677900. doi: 10.3389/fnut.2025.1677900 (PMC12671200; doi:10.3389/fnut.2025.1677900)
Supplement: Supplementary file 1 [file Data_Sheet_1.pdf]

Supplementary Material

Table A.1. Prevalence (%) of motives for dietary choices by diet type for sex, school level, residence and employment status (N = 1,350). Vegetarian – lacto-ovo-vegetarian.

		Health	Taste/ Preference	Animal Welfare	Environment Protection	Tradition	Food Quality	Family	Sports Performance	Social Aspects	No specific reason/Other
TOTALSAMPLE	Overview	46.4	22.6	4.1	1.6	9.6	7.0	1.1	0.8	0.1	6.4
	Omnivorous	46.9	23.8	1.4	1.1	10.2	7.4	1.1	0.9	0.3	6.8
	Vegetarian	37.1	10.0	40.0	5.7	NA	2.9	1.4	NA	1.4	1.4
	Vegan	48.4	3.2	29.0	12.9	3.2	NA	NA	NA	NA	3.2
MALE	Omnivorous	45.9	25.8	1.0	0.8	10.5	4.3	0.5	1.8	0.3	9.2
	Vegetarian	30.0	NA	50.0	10.0	NA	NA	NA	NA	NA	10.0
	Vegan	28.6	NA	57.1	14.3	NA	NA	NA	NA	NA	NA
FEMALE	Omnivorous	47.4	22.9	1.6	1.3	10.2	8.9	1.4	0.5	0.2	5.7
	Vegetarian	38.3	11.7	38.3	5.0	NA	3.3	1.7	NA	1.7	NA
	Vegan	54.2	4.2	20.8	12.5	4.2	NA	NA	NA	NA	4.2
MIDDLE SCHOOL	Omnivorous	50.0	21.9	1.4	0.5	11.3	7.0	1.4	1.0	NA	5.6
	Vegetarian	30.0	13.3	43.3	6.7	NA	NA	NA	NA	3.3	3.3
	Vegan	40.0	10.0	30.0	10.0	NA	NA	NA	NA	NA	10.0
HIGH SCHOOL	Omnivorous	46.3	23.2	1.0	1.0	10.9	8.5	0.3	0.8	0.2	7.8
	Vegetarian	55.6	7.4	25.9	7.4	NA	3.7	NA	NA	NA	NA
	Vegan	53.8	NA	23.1	15.4	7.7	NA	NA	NA	NA	NA
MIDDLE & HIGH SCHOOL OOLED	Omnivorous	43.2	28.4	2.5	NA	7.8	5.8	2.5	0.8	NA	6.5
	Vegetarian	15.4	7.7	61.5	NA	NA	7.7	7.7	NA	NA	NA
	Vegan	50.0	NA	37.5	12.5	NA	NA	NA	NA	NA	NA
URBAN	Omnivorous	44.5	25.1	1.5	1.5	8.7	8.1	0.9	0.7	0.2	8.7
	Vegetarian	38.2	5.9	38.2	5.9	NA	5.9	2.9	NA	NA	2.9
	Vegan	58.8	NA	29.4	5.9	NA	NA	NA	NA	NA	5.9
RURAL	Omnivorous	48.3	23.0	1.4	0.9	11.4	7.1	1.3	1.0	NA	5.7
	Vegetarian	36.1	13.9	41.7	5.6	NA	NA	NA	NA	2.8	NA
	Vegan	35.7	7.1	28.6	21.4	7.1	NA	NA	NA	NA	NA
FULL TIME	Omnivorous	48.1	24.2	1.2	1.0	10.2	6.5	0.9	0.9	0.1	6.7
	Vegetarian	36.7	10.2	38.8	6.1	NA	2.0	2.0	NA	2.0	2.0
	Vegan	37.5	4.2	37.5	12.5	4.2	NA	NA	NA	NA	4.2
PART TIME	Omnivorous	42.6	22.2	2.2	1.5	11.1	10.7	1.9	0.7	NA	7.0
	Vegetarian	38.1	9.5	42.9	4.8	NA	4.8	NA	NA	NA	NA
	Vegan	85.7	NA	NA	14.3	NA	NA	NA	NA	NA	NA

Note. NA – no answer/not applicable.

Table A.2. Prevalence (%) of lifestyle preferences for dietary choices by diet type for sex, school level, residence and employment status (N = 1,350). Vegetarian – lacto-ovo-vegetarian.

		Sport Engagement	Sport Lifestyle	Eating Meat	Vegetarian Diet	Vegetarian Lifestyle	Vegan Diet	Vegan Lifestyle	Alcohol	Smoking
TOTAL SAMPLE	Overview	70.7	10.9	4.1	7.4	2.4	1.1	0.9	1.6	0.7
	Omnivorous	73.7	11.4	4.5	5.0	2.2	0.1	0.6	1.8	0.7
	Vegetarian	31.4	5.7	NA	51.4	5.7	4.3	1.4	NA	NA
	Vegan	41.9	3.2	NA	3.2	3.2	35.5	9.7	NA	3.2
MALE	Omnivorous	79.1	10.5	3.6	3.1	1.3	NA	0.3	1.5	0.8
	Vegetarian	30.0	NA	NA	50.0	10.0	10.0	NA	NA	NA
	Vegan	71.4	NA	NA	NA	NA	14.3	14.3	NA	NA
FEMALE	Omnivorous	71.2	11.8	4.9	6.0	2.7	0.1	0.8	1.9	0.7
	Vegetarian	31.7	6.7	NA	51.7	6.7	3.3	NA	NA	NA
	Vegan	33.3	4.2	NA	4.2	4.2	41.7	8.3	NA	4.2
MIDDLE SCHOOL	Omnivorous	73.3	13.5	4.6	4.3	2.2	NA	0.5	0.7	1.0
	Vegetarian	23.3	NA	NA	60.0	10.0	3.3	3.3	NA	NA
	Vegan	30.0	NA	NA	NA	10.0	40.0	20.0	NA	NA
HIGH SCHOOL	Omnivorous	74.7	10.0	3.7	5.8	2.2	02	0.7	2.0	0.7
	Vegetarian	40.7	14.8	NA	33.3	3.7	7.4	NA	NA	NA
	Vegan	38.5	7.7	NA	7.7	NA	30.8	7.7	NA	7.7
MIDDLE & HIGH SCHOOL POOLED	Omnivorous	71.6	11.1	6.2	4.5	2.5	NA	0.8	2.9	0.4
	Vegetarian	30.8	NA	NA	69.2	NA	NA	NA	NA	NA
	Vegan	62.5	NA	NA		NA	37.5	NA	NA	NA
URBAN	Omnivorous	73.6	10.5	3.9	6.6	2.0	NA	0.4	2.0	1.1
	Vegetarian	32.4	8.8	NA	55.9	NA	2.9	NA	NA	NA
	Vegan	47.1	NA	NA	5.9	5.9	29.4	11.8	NA	NA
RURAL	Omnivorous	73.7	11.9	4.8	4.2	2.4	0.1	0.8	1.6	0.5
	Vegetarian	30.6	2.8	NA	47.2	11.1	5.6	2.8	NA	NA
	Vegan	35.7	7.1	NA	NA	NA	42.9	7.1	NA	7.1
FULL TIME	Omnivorous	73.4	12.0	4.8	4.9	2.2	NA	0.5	1.3	0.8
	Vegetarian	28.6	8.2	NA	51.0	8.2	2.0	2.0	NA	NA
	Vegan	41.7	NA	NA	NA	4.2	37.5	12.5	NA	4.2
PART TIME	Omnivorous	74.4	9.3	3.3	5.6	2.2	0.4	1.1	3.3	0.4
	Vegetarian	38.1	NA	NA	52.4	9.5	NA	NA	NA	NA
	Vegan	42.9	14.3	NA	14.3	NA	28.6	NA	NA	NA

Note. NA – no answer/not applicable.

This is a provisional file, not the final typeset article
